# Supplementary material for: Comparative outcomes of combined corticosteroid and remdesivir therapy with corticosteroid monotherapy in ventilated COVID-19 patients
Source: PLoS One. 2022 Feb 23;17(2):e0264301. doi: 10.1371/journal.pone.0264301 (PMC8865672; doi:10.1371/journal.pone.0264301)
Supplement: Attachment — Submitted filename: Response to reviewers.docx [file pone.0264301.s003.docx]

**Manuscript** PONE-D-21-08678R1

**Response to Reviewers**

Dear Editor,

We are thankful for allowing us to submit a revised draft of the manuscript "Comparative outcomes of combined corticosteroid and remdesivir therapy with corticosteroid monotherapy in ventilated COVID-19 patients" for publication in the reputed PLOS ONE scientific journal. We appreciate the time and effort you and the reviewers dedicated to providing feedback on our manuscript. We are grateful for the insightful comments guiding us to make valuable improvements to our paper and educate young clinical researchers like us. We have incorporated the suggestions made by the reviewers, and those changes are depicted under track changes within the manuscript word file. We acknowledge our errors raised by the reviewers and have added the required manuscript to the revised manuscript.

Please see below for a point-by-point response to the reviewers' comments and concerns.

**Reviewer # 2 comments:**

**1. Abstract: Sentence is staring with abbreviation. Please reveres that RDV (Remdesivir) to Remdesivir (RDV). Similarly need to be looked at other places as well.**

Thank you for the precise observation. These will be very helpful as reminders for our future work. We have made necessary corrections throughout the manuscript where necessary, including the abstract.

**2. All cause 28-day mortality rates between matched cohorts (28/28) calculated after five days of CSRDV and CS therapy were similar (51% vs. 70%; P=0.11). Although mortality is not showing statistical significance. However, difference is approximately 19%.**

We appreciate the reviewer reminding us to add necessary details to the manuscript, which are critical. We amended the sentence in the first paragraph of the discussion section as suggested. The new sentence added reads as: "The all-cause 28-day mortality rates between matched cohorts (28/28) calculated after five days of CS-RDV and CS therapy were 51% and 70%, respectively (P=0.11). The difference between the matched cohorts appears to be numerically significant at 19% but did not reach statistical significance, necessitating further evaluation with larger sample size."

**3. 'Patients with elevated transaminases more than five times the standard upper normal limit was excluded from the CSRDV study arm'. This is confusion. Whether authors have prospectively enrolled the patients or analyzed from database.**

We appreciate the reviewer educating us on crafting the sentences carefully to avoid confusion. We amended sentences 109 -111 in the first paragraph of the study population section as per the suggestion. The new sentence reads as: "While selecting patients retrospectively for the CS-RDV group, we did not include patients with elevated transaminases more than five times the standard normal upper limit."

**4. Line 137 Secondary bacterial infection write as SBI**

Thank you for the feedback. We have made necessary corrections throughout the manuscript where necessary.

**5. Authors have used both Covid-19 as well as COVID-19. To maintain uniformity one abbreviation should be used. Similarly, syntax and grammar need to be looked throughout the manuscript**

We apologize for our error. We have made necessary corrections throughout the manuscript where necessary.

Sincerely,

Subhadra Mandadi. MD.

Corresponding author.

Date: 2/1/22
